# Crustal Density Structure of the Jiuzhaigou Ms7.0 Earthquake Area Revealed by the Barkam–Jiuzhaigou–Wuqi Gravity Profile

**DOI:** 10.3390/s21041497

**Published:** 2021-02-21

**Authors:** Guangliang Yang, Chongyang Shen, Hongbo Tan, Jiapei Wang

**Affiliations:** 1 Key Laboratory of Earthquake Geodesy, Institute of Seismology, China Earthquake Administration, Wuhan 430071, China; vforyang@gmail.com (G.Y.); thbhong@163.com (H.T.); wang_jia_pei@163.com (J.W.); 2 Institute of Disaster Prevention, Langfang 065201, China; 3 State Key Laboratory of Geodesy and Earth’s Dynamics, Innovation Academy for Precision Measurement Science and Technology, Chinese Academy of Sciences, Wuhan 430077, China; 4 Wuhan Gravitation and Solid Earth Tides National Observation and Research Station, Wuhan 430071, China

**Keywords:** Jiuzhaigou Ms7.0 earthquake, gravity profile, crustal structure, gravity anomaly

## Abstract

The Barkam–Jiuzhaigou–Wuqi gravity profile extends across the Jiuzhaigou Ms7.0 earthquake (in 2017) zone and passes through several historical big earthquakes’ zones. We have obtained Bouguer gravity anomalies along the profile composed of 365 gravity observation stations with Global Positioning System (GPS) coordinates, analyzed the observed data and inverted subsurface density structure. The results show that the Moho depth has a big lateral variation from southwest to northeast, which shallows from 57 km to 43 km with maximum variation up to 14 km within 800 km. The most acute depth change of the Moho is in the boundary region between the Bayan Har block and West Qinling–Qilian block. According to our analysis, it is related to the eastward movement of the Bayan Har block. There are three main pieces of evidence that support it: (1) Density is higher in the east of the Bayan Har block and smaller in the west, which is the same as seismic activity; (2) Two thin low-density layers exist in the upper and middle crust of the Bayan Har block, which may promote inter-layer slip and the Jiuzhaigou Ms7.0 earthquake occurred in the boundary area of the two low-density layers, where the crustal density and Moho surface fluctuate sharply; (3) the GPS velocity field in the southwestern part gravity profile is significantly larger than that of the northeastern part, which is consistent with the density structure. Our studies also suggest that the large undulation of the Moho prevents the movement of the Bayan Har block, and strain is prone to accumulate here. The dynamic background analysis of the crust in this area indicates that the Moho surface uplifts in the West Qinling–Qilian block, which decelerates the eastern migration of material on the Qinghai–Tibet Plateau, and leads to the weak tectonic activity of the north part of the Bayan Har block.

## 1. Introduction

**Figure 1:**
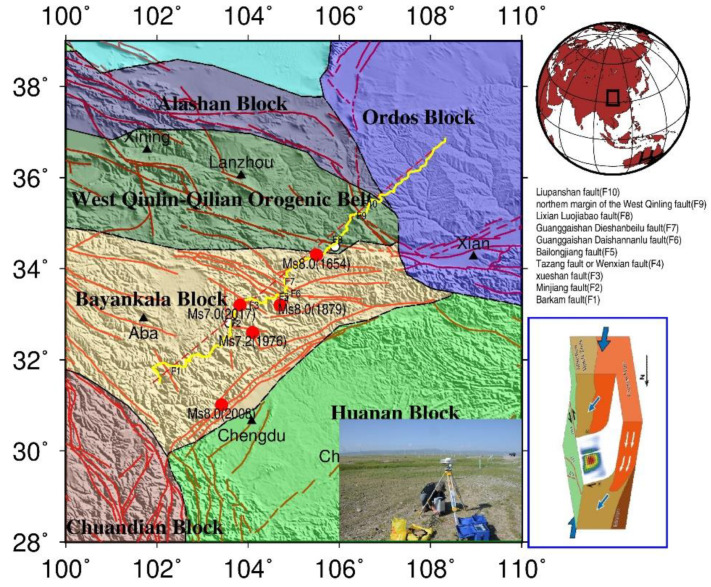
Distribution of gravity/GPS stations and fault in the Ms7.0 earthquake area in Jiuzhaigou, Sichuan Province. The yellow points represent the distribution of gravity and GPS stations; the red lines represent faults [19]; the white circles represent absolute gravity station; the black triangles represent place name; the red circles represent the epicenters of Ms7.0 or larger earthquake in the area, data from China Earthquake Networks Center; the photo in this figure is a field observation map; and the rectangle in insert map in the lower right corner represents the study area. The fault names of F1~F10 are displayed in the middle right of this figure, F1 represents Barkam fault, F2 represents the Minjiang fault, F3 represents the Xueshan fault, F4 represents the Tazang fault (Wenxian fault), F5 represents the Bailongjiang fault, F6 represents the Guanggaishan-Daishannanlu fault, F7 represents the Guanggaishan–Dieshanbeilu fault, F8 represents the Lixian-Luojiabao fault, F9 represents the northern margin of the West Qinling fault, F10 represents the Liupanshan fault. The tectonic movement background picture on the lower right of this figure is from [7].

In the past two decades, big earthquakes have been occurring more frequently in the Sichuan–Yunnan region, especially near the eastern and northern boundaries of the Bayan Har block on the northeastern edge of the Qinghai–Tibet Plateau, such as the Ms7.9 Mani earthquake in 1997, –s8.1 West Kunlun Mountain earthquake in 2001, Ms8.0 Wenchuan earthquake in 2008, Ms7.1 Yushu earthquake in 2010, and Ms7.0 Lushan earthquake in 2013, and these earthquakes show a tendency to migrate to the eastern boundary of the Bayan Har block. The Ms7.0 Jiuzhaigou earthquake [1] in 2017 is the latest event (Figure 1). This earthquake occurred on the east of the Eastern Kunlun fault belt of the eastern Bayan Har block, where the Tazang fault, Minjiang fault, and Huya fault converge toward together and is approximately 20 km [2,3,4,5] beneath the crust. This area is also considered to be the triple junction of the Minjiang tectonic belt, Longmenshan orogenic belt, and the West Qinling–Qilian orogenic belt. The focal mechanism shows that the Jiuzhaigou earthquake is dominated by left-lateral strike-slip faulting [5,6,7], which is different from surrounding dip-thrust earthquakes.

There are many questions that need to be further clarified in this region, e.g., how does the subsurface matter of the Qinghai–Tibet Plateau move? How do the blocks interact with each other? How does the follow-up earthquake develop? The crustal density structure records the long-term evolution of crustal tectonic movement. We hope to obtain clues to the above problems through the high-precision density distribution of block boundaries and faults and provide insights of the tectonic evolution and seismic development trends in the region.

There are many geophysical studies in this area [8,9,10,11,12], for instance, surface waves, deep reflection seismic profile, magnetotelluric (MT). However, geodetic field observations are very sparse because of the harsh natural environment, high altitude, and poor traffic. Studies on the crustal structure and tectonic movement are not quite clear. The results of seismic imaging reveal [8,10] that the depth of the Moho surface is between 48 and 51 km with the northern part deeper than the southern part. The inter-structure of the crust varies significantly not only in vertical but also in horizontal, and the thicknesses of Moho in the eastern and western regions are quite different. Seismic imaging shows low-velocity layer structure; however, the spatial resolution is more than tens of kilometers, so it is hard to depict the deep structure features precisely.

Gravity inversion method is considered one of the important geophysical tools in exploring the gravity anomalies caused by the subsurface density disturbance [13,14,15,16,17,18] and has strong horizontal resolution compared with seismic method. These methods can also be constrained by other geophysical data sources, such as seismic, magnetotelluric (MT) and achieve a consistent crustal structure. So, gravity methods have the advantages of providing crustal horizontal undulation, especially for the fluctuation of the Moho surface, density distribution, fold-and-thrust belt geometry, low-density body (abnormal density body), and crustal tectonic properties. All these density structures are related to crustal tectonic movement and seismic activity.

To analyze the geometric distribution of the precise crustal density in the Jiuzhaigou area, the tectonic relationships among several adjacent blocks, and the deep tectonic setting and dynamic mechanism of the epicenter area, we observed the gravity and GPS coordinate data. By constructing a precise layered crustal structure along this gravity profile, we have obtained difference of the crustal density in depth and different parts of blocks. These study results will provide support for the mechanism of seismic processing, information on the eastward movement of the Qinghai–Tibet Plateau, and the uplift of the eastern margin of Bayan Har block in the Qinghai–Tibet Plateau.

## 2. Data

The Barkam–Jiuzhaigou–Wuqi gravity profile is shown in Figure 1 (Line composed of yellow dots). Data are collected from 365 sites (gravity and GPS) distributed across the 1976 Songpan Ms7.2 earthquake area, 1879 Wudu Ms8.0 earthquake area and 1654 Tianshui Ms8.0 earthquake area from Barkam City, Sichuan Province, to Wuqi Town, Shanxi Province. The profile is about 800 km long. The average distance between measuring stations is approximately 2.5 km, and the maximum distance between stations is no more than 3 km. Gravity and GPS coordinate observations are performed simultaneously at every position. Gravity measurements are obtained using CG-5 gravimeters with a resolution of 0.01 mGal (mGal = 10^−8^ m·s^−2^, the same below) and an accuracy of ± (0.02–0.03) mGal. Two absolute gravity stations are located at Tianshui and Songpan. GPS coordinates are obtained using Trimble 5700 receiver and zephyr geodetic antenna and each station is observed for at least 40 min with a sampling rate of 30 s. The gravity-measuring stations and faults in this region are shown in Figure 1.

The study area is in the northeastern margin of the Qinghai–Tibet Plateau and the boundary belt of several large tectonic blocks. Since the Wenchuan Ms8.0 earthquake, it has been attracting considerable interest. The tectonic structure is severe and complex because crustal movements on the eastern margin of the Qinghai–Tibet Plateau are affected by the subduction and collision of the Indian Plate and the blockage of the Sichuan Basin [5,7]. There are three tectonic units from west to east according to seismic activity: the Bayan Har block, Longmenshan orogenic belt, and the Sichuan Basin. It can also be divided into three blocks from south to north including the Bayan Har block, West Qinling orogenic belt and Ordos block [8,9].

GPS data are processed using the software GIPSY (version 6.2) [20] with precise single-point positioning mode, considered ionospheric model, tropospheric correction, tide correction model, etc., to improve positioning accuracy. The processing results show that the horizontal accuracies of GPS measurements are generally within 15 cm and the elevation accuracies are all within 30 cm.

**Figure 2:**
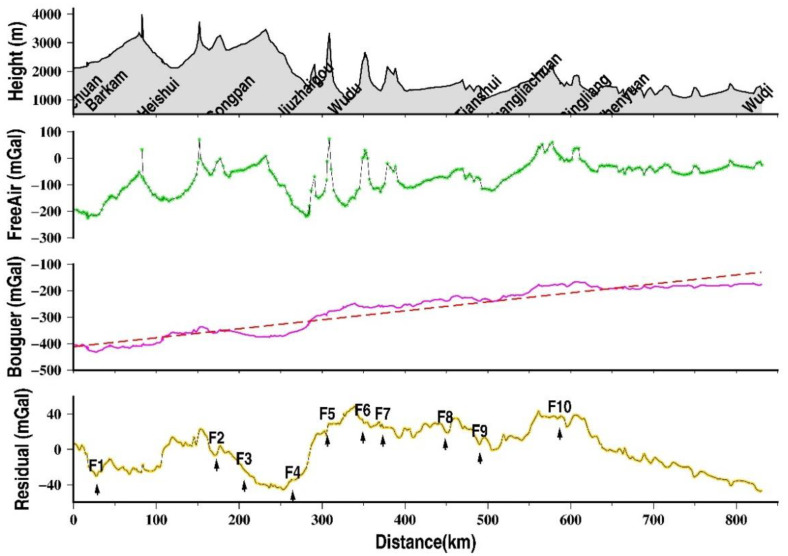
Topography, free-air anomalies, Bouguer anomalies and residual anomalies along with the Barkam–Jiuzhaigou–Wuqi profile (from top to bottom). In the last graph, F1~F10 represents fault, same as Figure 1.

The observed gravity data are reduced and adjusted successively, such as tidal, barometric, displacement, instrumental height. Gravity adjustment is constrained by absolute gravity stations in Tianshui and Songpan (white circle in Figure 1). The free-air gravity anomalies of the profile have undergone a series of gravity corrections, including the normal field correction, height correction, Bouguer plate correction and terrain correction with a maximum radius of 166.7 km using a topographic density of 2.67 g/cm^3^ based on ASTER GDEM (Advanced Spaceborne Thermal Emission and Reflection Radiometer Global Digital Elevation Model) version 2 data [21] and GPS surveys. The formulae for these calculations refer to [22]. The complete Bouguer gravity anomalies after the above correction are as shown in Figure 2.

From Figure 2, the free-air gravity anomalies range from −226 mGal to +52 mGal with an average of −100 mGal, which correspond well with the terrain from the southwest to the northeast. Maximum and minimum anomalies appear to coincide with mountains and valleys, especially in Barkam, Songpan, Jiuzhaigou, and Wudu, etc. The Bouguer gravity anomalies range from −433 mGal to −175 mGal and show an uplift trend from the southwest to the northeast. In the southwest part of the profile (Barkam to Wudu), the gravity varies greatly, then changes slowly in the middle part, which is a transitional area, and finally tends to be stable in the northeastern part. The Bouguer gravity anomalies are also clearly related to the faults (F1~F10 in Figure 2). From southwest to northeast, this profile successively passes through the Barkam fault (F1), MingJiang fault (F2), Xueshan fault (F3), Tazhang fault (F4), Bailongjiang fault (F5), Guanggaishan-Daishannanlu fault (F6), Guanggaishan–DaishanBeilu fault (F7), Lixian–Luojiapu fault (F8), Northern margin of the West Qinling fault (F9), and Liupanshan fault (F10). Gravity disturbances in the Barkam, Lancangjiang, Xueshan, Tazhang, Lixian–Luojiapu, and Diebu–Bailongjiang fault zones are obvious. Furthermore, the Bouguer gravity anomalies and residual anomalies (removed trends) of the profile illustrate segmental features. It could be considered that there are three different density structures along this profile, which are divided by the Tazang fault and the Lixian–Loujiapu fault. For example, magnitude and variation of gravity anomalies are characterized by segmentation, and detailed descriptions are shown in Table 1: The Bouguer gravity anomalies change intensely in the Jinchuan–Jiuzhaigou part (crustal fold belt area), but slowly in the Jiuzhaigou–Pingliang part (transition area) and nearly stable in the Pingliang–Wuqi part (Craton area). The segmental feature of gravity anomalies is basically consistent with the geological block boundary division in the Bayan Har block and the Ordos block [19].

The detailed Bouguer gravity anomalies of each segment are as follows: from Jinchuan to Jiuzhaigou, the gravity anomalies varied from −433 mGal to −280 mGal with uneven, irregular changes, and there is a local low values region of Bouguer anomalies from 60 to 100 mGal across an approximately 100 km width between Songpan and Jiuzhaigou. From Jiuzhaigou to Pingliang, the gravity anomalies increase linearly, and the overall change is relatively small, ranging from −292 mGal to −211 mGal. Local rapid variations of the Bouguer gravity anomalies appear in near Tianshui, especially in the northern margin of the Qinlian fault, and the maximum change reaches 30 mGal. From Pingliang to Wuqi, the gravity anomalies are relatively flat and show a small change with an average of approximately −183 mGal, which is consistent with the characteristics of the Ordos block.

To sum up, the boundaries of adjacent blocks can be significantly identified by the Bouguer gravity anomalies in this profile. The Bouguer gravity anomalies of the 284 km Bayan Har block profile vary from −443.2 to −331 mGal, with an average of −383 mGal, which is 0.395 mGal/km. As for the West Qinling orogenic belt, the average anomaly is approximate −274.4 mGal (378.6~−170.2 mGal), and the change rate reaches 0.661 mGal/km with the length of 316 km. However, the average anomaly of the Ordos block is −177 mGal (−181.5~−163.4 mGal), and the change rate of gravity anomalies is 0.079 mGal/km, which is far less than the other two parts (see Table 1). We need to pay special attention to these places where the most rapid changes of the Bouguer gravity appear, such as Heishui, Songpan, Jiuzhaigou, Tianshui. Gravity changes reflect changes of density inside the earth’s crust. That is, in the study area, huge density changes are strongly correlated with large earthquakes.

## 3. Crustal Model and Density Structure

### Initial Crustal Model

To obtain the internal density structure of the earth’s crust and seismic-related tectonic background, we tried to invert the internal structure of the earth’s crust using the Bouguer anomaly described above. As we know, potential field methods like gravity suffer significantly from non-uniqueness, which usually cannot determine both the geometric shape and its density. However, the geophysical anomaly is homologous. Density anomalies zone is also the area where the seismic velocity, resistivity, and other geophysical observations appear abnormal. To reduce the non-uniqueness, we introduce the initial model constrained by the results of deep seismic reflection, earthquake imaging and magnetotelluric (MT).

There are several published crustal models in our study region. These models are constructed either from wide-angle deep reflection, P-wave receiver functions earthquake, surface wave tomography or ambient noise tomography [8,9,10,11,12,23,24,25]. The study of the P-wave receiver functions of tele-seismic data shows that multiple low-speed, low-density belts exist in the upper and middle crust below the plateau from the Bayan Har block to the central-southern Longmenshan region at depths of approximately 10~15 km and 24–45 km [23,24,25], which is consistent with the results of Aba-Wuqi deep seismic reflection profile (from project report of the geophysical exploration center, China earthquake administration). Depth of crustal layers and Moho surface are obtained by Aba-Wuqi deep seismic reflection profile, Heishui–Santai profile, and Zhubalong–Zizhong profile [26]. There is little difference in the Moho interface compared with the average thickness of the Bayan Har block, approximately 60–62 km. The initial model is constructed based on the seismic velocity results in this area [8,9,10,11,12,23,24,25,26]. Based on the above geophysical observations and velocity/density conversion [27], an initial model of the crustal structure is obtained and shown in Table 2.

**Figure 3:**
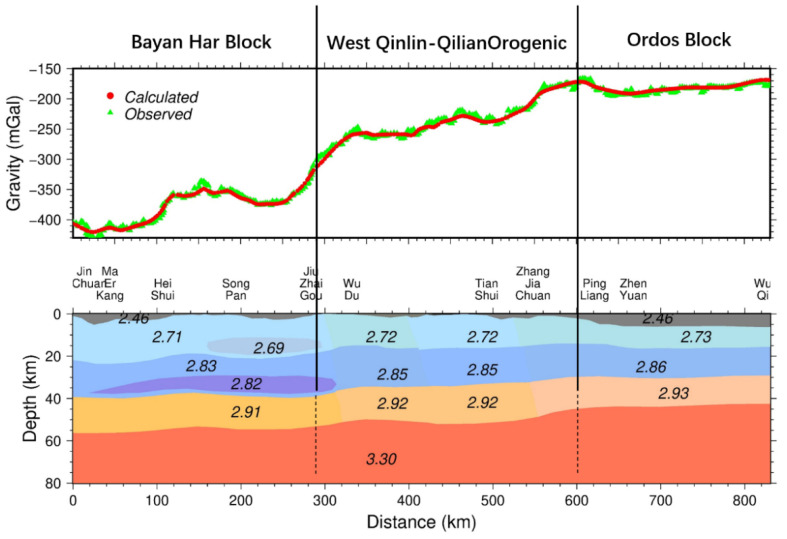
The crustal density structure along with the Barkam–Wuqi profile. The upper figure shows observed gravity anomalies and calculated gravity anomalies, which is derived from the forward calculation of density anomalies in the lower figure. The black lines represent a block boundary, where there is a greater lateral difference in density, and the dotted line indicates that the information of the lower crust is uncertain. The numbers in the lower figure represent crust density (unit: g/cm^3^).

Interactive modeling [28] of the Bouguer anomaly, or trial-and-error, is performed using the free version of gm-sys software [29]. Firstly, we adjust Crustal stratification and/or its density combined with seismic and electromagnetic results. Secondly, we compare the deviation between the forward and the observed gravity values. Combining the abovementioned existing geophysical results, we adjust the depth of crustal layer and density repeatedly within a certain range and then obtain a density structure. If the density structure is basically consistent with the geophysical observations and the observed gravity anomalies are consistent with that caused by the subsurface density disturbance, we obtain the density structure along the profile (shown in Figure 3). So, the density structure model obtained by our calculation (trial-and-error) is consistent with the observed gravity anomalies, and consistent with the existing seismic velocity, electrical structure results and geological cognition in the area.

The density structure of Figure 3 consists of four layers in the vertical direction: the low-density cap layer (sedimentary cap rocks), upper crust, middle crust, and lower crust. The thickness of the low-density cap layer is approximately 0–6 km with an average of 2 km, and the density is approximately 2.46 g/cm^3^. The cap layer of the southwestern Bayan Har block is thin, whereas the northeastern part near the Ordos shows gradually thickening. The depth of the upper crust is 18–22 km with an average of 20 km, and the density is 2.71~2.73 g/cm^3^. A local low-density body with a density of 2.69 g/cm^3^ appears in the southwest of the Bayan Har block. The overall density of the southwestern part is extremely low, showing that the crustal movement is active. The depth of the middle crust ranges from 29.5 to 42.3 km, the average value is 35 km, and the density is 2.83~2.86 g/cm^3^. The southwestern Bayan Har block is a low-density body with a density of 2.82 g/cm^3^. The depth of the Moho surface is 45.5–56.5 km with an average of 53 km, the fluctuation is large, and the density of the lower crust ranges within 2.91–2.93 g/cm^3^.

In the orthogonal direction of the profile, it is characterized by distinct subblock. The discrepancies between the three blocks, Bayan Har block, West Qinling–Qilian orogenic belt, Ordos block are obvious (see Table 3 for details.) in the following several aspects.

(1) In the upper crust and the middle crust of the southwestern part of the Bayan Har block, there are two local low-density bodies with thin thickness, whereas they disappear in other blocks.

(2) From the statistics of the average depth and variation range of cover layer, the upper, middle, and lower crust layer of Bayan Har block, West Qinling–Qilian organic belt and Ordos block in Table 2, the results show that the depth decreases with rising interface from the Bayan Har block, West Qinling–Qilian orogenic belt, to Ordos block.

(3) From the perspective of density distribution, density gradually increases from southwest to northeast except for low density, thin layer. However, the discrepancy of the density is not obvious. As for the fluctuation of crustal layer interfaces, most intense for the West Qinling–Qilian orogenic belt, as a fold-thrust belt which is a boundary transition zone, followed by Bayan Har block, less for Ordos block.

## 4. Discussion

### 4.1. Comparison with Crust1.0 models

From Figure 3 and Table 3, we propose that a crustal profile model obtained from gravity interactive inversion not only reveals crustal thickness variation and Moho geometry but also shows the distinctive density discrepancies. For example, the thin crust in the southeast, thick crust in the northeast and transition in central mark the different tectonic units between the overthrust or folding and collision; the two low-densities’ anomalies exist beneath the Bayan Har block in the middle and upper crust, respectively. The spatial variations of the crustal thickness/density model show a strong lateral change in the upper crust, middle crust, and Moho topography.

**Figure 4:**
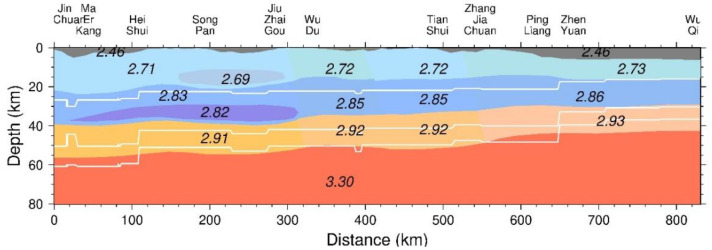
Crustal structure density model of gravity studies and Crust1.0 (the white lines represent upper crust, middle crust, and lower crust, respectively.) The numbers in the figure represent crust density (unit: g/cm^3^). Crust1.0 model data from [31].

In general, compared with Crust1.0 [30,31], obtained from active source seismic studies as well as from receiver function studies (Figure 4), spatial variation of the crustal density structures and Moho boundary obtained from our gravity study is in good agreement with previous studies [8,9,10,11,12,23,24,25,26]. The similarity of interface’s depth between our model and Crust1.0 indicates the robustness of our result. As can be seen from Figure 4, the improvement of our model in resolution is especially clear at the interface undulation, layers depth, and density distribution. However, previous regional studies have less details rather than rough layer undulation as in the Crust1.0 model. It is worth mentioning that the results clearly characterize the spatial geometry of the low-density layer constrained by rough low-speed layer depth and velocity information of seismic imaging. Although the initial model refers to the results of the seismic velocity, electrical structure results and geological cognition, the results obtain a more detailed model of the crustal density structure, which was not available before, and are in a good agreement with seismic and magnetotelluric models [10,11,12].

### 4.2. Relationship between GPS Velocity Field and Crustal Density

**Figure 5:**
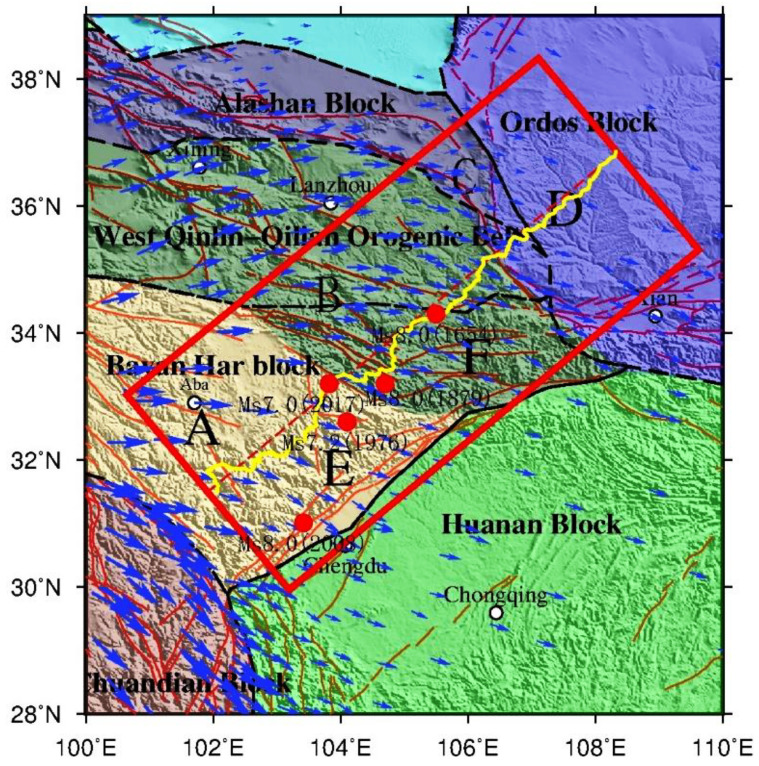
Velocity field of GPS observation and big earthquakes in the study area. The blue arrows represent the velocity field of GPS observation from [32]; the red rectangle denotes the study area; the yellow line represents gravity profile; the red circle represents the big earthquake (greater or equal to Ms8.0) that occurred in this area in history.

The main features obtained in our study can also be traced in the continental-scale tectonic movement. Almost all the geophysical phenomena in the Tibetan Plateau are related to mass migration. In the context of the Qinghai–Tibet Plateau as it moves eastward, thin low-density anomalies may promote interlayer slip. One of the obvious pieces of evidence is that the GPS velocity field (Figure 5) in the southwestern part (A, E in Figure 5) is significantly larger than that of the northeastern part (C, D in Figure 5), which is consistent with the density structure. In this study area (red rectangle of Figure 5), GPS speed of zone A is greater than zone E, zone B is greater than zone F, and zone C is greater than zone D in the direction orthogonal to the profile. The closer to the Longmenshan fault zone, the smaller the speed of the GPS velocity field. Along the profile, GPS velocities gradually become smaller from southwest to northeast. The lateral variation of the GPS velocity field is basically consistent with the variation characteristics of crustal density. The cause of this feature should be related to the crustal structure. One possible explanation is that the two low-density thin layers in the middle and upper crust of the Bayan Har block may promote the movement between the layers, which make the southwestern material (A, E) easier to move. Therefore, a stress accumulation region is formed at the end of the low-density body, that is, a high-risk area.

### 4.3. Relationship between Crustal Structure and Earthquakes

As mentioned above, this profile crosses the historical great earthquake zone (earthquakes that are equal to or greater than Ms7.0), such as the 1976 Ms7.2 Songpan earthquake, the 2017 Ms7.0 Jiuzhaigou earthquake, the 1879 Ms8.0 Wudu earthquake and the 1654 Ms8.0 Tianshui earthquake (red circle in Figure 5). From the time and space distribution of these great earthquakes, it shows that the epicenter tends to transfer from north to south. Furthermore, from the GPS observations mentioned above, the crustal movement rate gradually decreases from the southwest to the northeast. These results are basically consistent with the density distribution, showing for each layer a gradual increase along the profile. Therefore, we can consider that the Bayan Har block tends to migrate to the east and gets gradually shorter.

The Jiuzhaigou earthquake occurred at the boundary between the Bayan Har block and the West Qinling–Qilian orogenic belt with different movement speeds and near the Tazang fault, where the Moho surface fluctuates significantly. The large undulations of the Moho surface provide a strong barrier to block motion, and thus creates a condition for stress accumulation [6] that ultimately leads to the occurrence of earthquakes. As mentioned above, the Ms7.0 Jiuzhaigou earthquake is highly correlated with the Huya fault with a left-lateral strike-slip and fault rupture direction is consistent with the block boundary [33,34,35,36]. So, the Jiuzhaigou earthquake should be the performance of the Bayan Kala block moving eastward. Generally, the rupture surface of the strike-slip fracture of the block boundary is very deep.

This indicates that the tectonic activity in the northeastern part of the Bayan Har block became weaker, and the block boundary shifted to the south. This may be related to the deformation (uplift) of the Moho surface caused by the deep mantle, which hinders the eastward movement of the material on the Tibetan Plateau. However, we need further observation and research to verify this assumption.

## 5. Conclusions

Large earthquakes often occurred in the eastern part of the Bayan Har block and its adjacent areas. To study the tectonic background and density structure, we observed gravity and GPS data simultaneously and obtained the Barkam–Jiuzhaigou–Wuqi gravity detection profile consisting of 365 measuring stations, through the study of the Bouguer gravity anomaly characteristics of the profile and the spatial distribution of the layered crustal density structure. We find evidence of the migration and convergence of the Bayan Har block to the east based on gravity profile, which is basically consistent with the GPS observation results and the trend of seismic activity. At the same time, the main possible causes of the Jiuzhaigou earthquake are obtained. The results are in a good agreement with seismic models and allow to make the following conclusions.

(1) The gravity profile crosses a cluster of large historic earthquakes, and the gravity anomaly changes dramatically from −433 mGal to −280 mGal. The crustal density distribution obtained by the seismic velocity structure shows that the crustal density structure exhibits layer vertically and horizontally. The differences between blocks units are obvious, and the small-scale changes near the tectonic boundary zones are severe. The Moho surface is in the range of 43–57 km, becoming shallower rapidly from southwest to northeast, such that the Moho surface changes 14 km within approximately 800 km. The change of the Moho surface mainly locates in the Bayan Har block and the West Qinling–Qilian block. The Ordos block retains craton state, and the crustal structure is stable.

(2) Against the background of the collision between the Indian Plate and the Eurasian Plate, powerful external forces promote interaction and movement among the blocks, resulting in strong crustal deformation in the study area. The density structure obtained from the gravity profile is consistent with the motion characteristics of the GPS velocity field. The analysis shows that the multilayer low-density bodies in the middle and upper crusts of the eastern Bayan Har block may promote interlayer slip. This slip causes the eastward migration of the upper part of the Bayan Har block, which is one of the reasons why the migration velocities between adjacent blocks on the eastern margin of the Qinghai–Tibet Plateau are inconsistent, as indicated by the GPS velocity field.

(3) The Jiuzhaigou earthquake occurred at the end of the East Kunlun fault, which is the boundary between the Bayan Har block and the West Qinling–Qilian orogenic belt, where the gravity anomalies change greatly. From earthquake precise positioning, it shows that the fault rupture of the Jiuzhaigou earthquake is consistent with the block boundary direction. The Jiuzhaigou earthquake developed in the boundary zone of the larger block, the velocity of the block movement is different, and the Moho surface had large fluctuations. The large undulations of the Moho surface strongly hindered block motions and provided structural conditions for the rapid accumulation of stress.

## Figures and Tables

**Table 1:** Bouguer gravity anomaly features of different parts of the Barkam–Jiuzhaigou–Wuqi profile (Unit: mGal).

Bayan Har Block	West Qinling–Qilian Orogenic Belt	Ordos Block
Average: −383.0	Average: −274.4	Average: −177.0
(−443.2~−331.0)	(−378.6~−170.2)	(−181.5~−163.4)
Ratio: 0.395 mGal/km	Ratio: 0.661 mGal/km	Ratio: 0.079 mGal/km

**Table 2:** Initial model of crustal structure.

Crust	Depth	P Wave Velocity	Low-Velocity Body/Layer
Upper crust	5~10 km	5.90 km/s	/
	15~22 km	5.85 km/s	Low-speed layer
	~30 km	6.18 km/s	/
Middle crust	38~42 km	6.50 km/s	Low-speed layer
Lower crust	48~58 km	6.80 km/s	/

**Table 3:** Comparison of the crustal structures of different blocks along with the Barkam–Wuqi profile.

	Bayan Har Block Unit: km	West Qinling–Qilian Orogen Unit: km	Ordos Block Unit: km
The bottom boundary of the cover layer	Average: 2.2	Average: 1.1	Average: 2.9
	(0.4~5.1)	(0.4~2.4)	(1.1~5.1)
The bottom boundary of the upper crust	Average: 20.8	Average: 14.8	Average: 12.2
	(18.4~23.5)	(12.8~16.6)	(11.1~12.8)
The bottom boundary of the middle crust	Average: 38.8	Average: 35.4	Average: 26.6
	(37.2~39.9)	(28.4~39.8)	(24.4~28.5)
The bottom of the lower crust	Average: 54.7	Average: 49.6	Average: 46.5
	(53.2~56.5)	(45.8~52.3)	(48.3~45.0)

## Data Availability

The data presented in this study are available on request from the corresponding author.
